# A New Genus of Aplodontid Rodent (Mammalia, Rodentia) from the Late Oligocene of Northern Junggar Basin, China

**DOI:** 10.1371/journal.pone.0052625

**Published:** 2013-01-24

**Authors:** Shundong Bi, Jin Meng, Sarah McLean, Wenyu Wu, Xijun Ni, Jie Ye

**Affiliations:** 1 Key Laboratory of Evolutionary Systematics of Vertebrates, Institute of Vertebrate Paleontology and Paleoanthropology, Chinese Academy of Sciences, Beijing, China; 2 Department of Biology, Indiana University of Pennsylvania, Indiana, Pennsylvania, United States of America; 3 Division of Paleontology, American Museum of Natural History, New York, United States of America; Monash University, Australia

## Abstract

A new genus and species of aplodontid rodent, *Proansomys dureensis*, from the late Oligocene of the northern Junggar Basin of China is described. The new genus is referred to as Ansomyinae because the ectoloph on the upper cheek teeth, although not fully crested, has attained the same characteristic bucket-handle-shaped configuration as other members of the subfamily. It represents the earliest record of the subfamily yet discovered in Asia and is more plesiomorphic than species of the genus *Ansomys* in having a partly crested ectoloph, a lower degree of lophodonty, and less complex tooth basins (lacking accessory lophules). *Proansomys* has transitional features between *Prosciurus* and *Ansomys*, suggesting that the Ansomyinae derived from a group of aplodontids related to *Prosciurus*, as did other advanced aplodontid rodents. This provides new light on the paleobiogeography of the Ansomyinae.

## Introduction

Mountain beavers of the subfamily Ansomyinae are small-sized aplodontid rodents, characterized by a bucket-handle shaped ectoloph on their upper cheek teeth (in occlusal view the buccal margin of the ectoloph undulates as does the grip on the handle of a bucket). Until recently the subfamily comprised only the type genus *Ansomys* and was found primarily from the late Oligocene and the middle Miocene of Eurasia [1], [2], [3], [4]. In the last few years, however, several new taxa have been discovered from the Oligocene and Miocene of North America [5], [6], [7]. Though found across the Holarctic region, the evolutionary origins and biogeography of the group are still poorly known because of relatively few records and poor representation of some taxa [5]. In this study, we describe a new genus and species of the basal subfamily Ansomyinae from the late Oligocene Tieersihabahe and Saerduoyila localities in northern Junggar basin of China. The specimens represent the earliest record of the subfamily in Asia and provide new information on the early history of the Ansomyinae.

**Figure 1:**
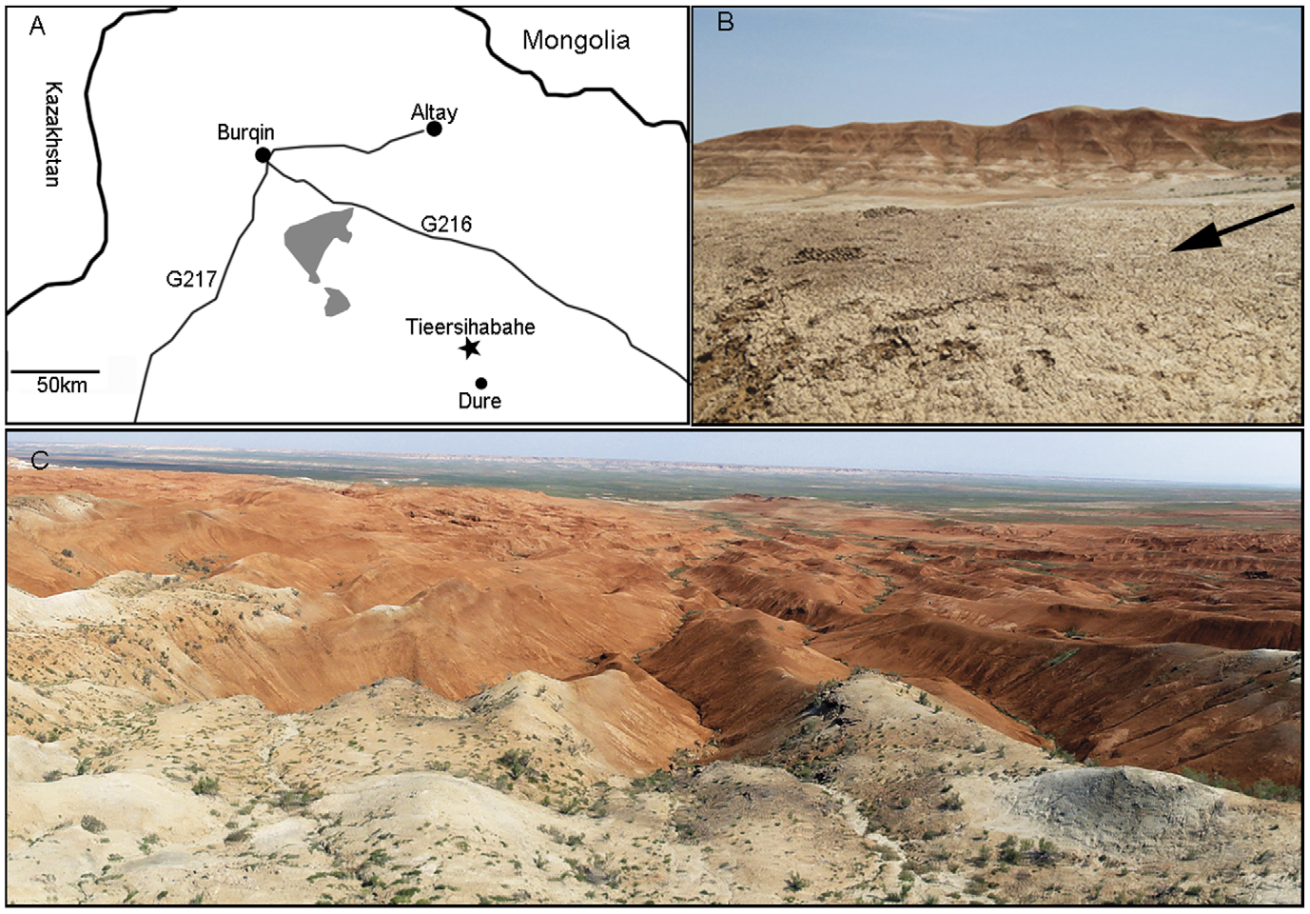
Location and overview of the Tieersihabahe locality. A, Location of the Tieersihabahe and Saerduoyila localities; B, close up of Tieersihabahe Formation where the fossils were found; C, Broad expanse of Tieersihabahe Section in the northern Junggar Basin. The black arrow in B above indicates the Tieersihabahe Formation.

**Figure 2:**
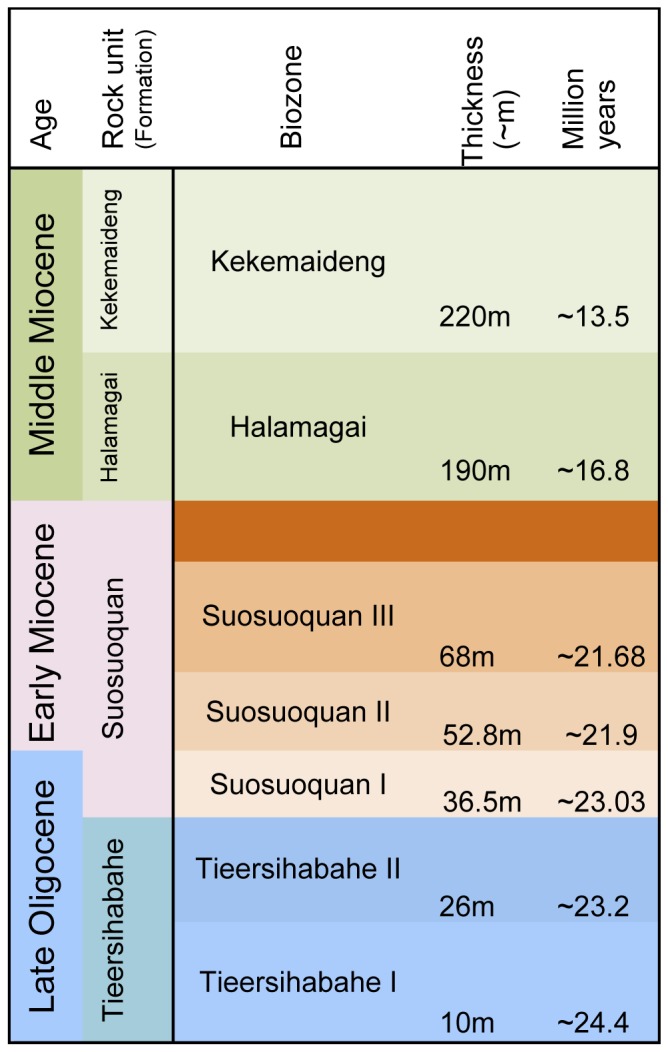
A summary of stratigraphic positions and age estimates for major mammal assemblages of Tieersihabahe Section in the northern Junggar Basin, China.

Tieersihabahe section in northern Junggar basin of China is renowned for its continuous sequences from the late Oligocene to middle Miocene that contains the Oligocene/Miocene boundary (Figure 1A–C). The fauna and stratigraphy have been widely studied by a number of researchers [8], [9], [10], [11], [12], and Meng et al. [13] presented a comprehensive biostratigraphy and magnetostratigraphy of the section and recognized five mammal assemblage zones for the lower Tieersihabahe and Suosuoquan formations. The biozones are, in ascending order from the older to younger, Tieersihabahe faunal assemblage Zones I and II, Suosuoquan faunal assemblage Zones I, II, and III (Figure 2). The majority of specimens described here, collected during the 1998, 1999, 2000, 2002, and 2004 expeditions, were from the Tieersihabahe faunal assemblage Zone I (T-I) and were identified as Ansomyinae gen.et sp. nov in faunal lists by Ye et al. [10]. The age of the fauna is biostratigraphically and paleomagnetically correlative to the late Oligocene of 24.4∼24.15 Ma [13]. The fossils were also found in 2000 from Saerduoyila, approximately 50 km northwest of Tieersihabahe section in the Halamagai area. The constitution of Saerduoyila fauna suggests that it was equivalent to the Tieersihabahe faunal assemblage Zone I.

## Materials and Methods

The material was obtained by surface collecting and screen washing from sites XJ98023, XJ98035, XJ200208, XJ200209, XJ200207, and XJ20004. All these sites except XJ20004 of Saerduoyila are from the Tieersihabahe section. Based on stratigraphic and faunal correlations, XJ98023, XJ98035, XJ200208, XJ200209, and XJ20004 are in the same horizon (Tieersihabahe faunal assemblage Zone I) of the Tieersihabahe Formation, and XJ200207 is in the Suosuoquan faunal assemblage Zone I of the Suosuoquan Formation, 20 m above Tieersihabahe faunal assemblage Zone I (Figure 2). All specimens are deposited in the collections of the Institute of Vertebrate Paleontology and Paleoanthropology (IVPP), Chinese Academy of Sciences, Beijing.

Dental terminology used follows that of [14]. Teeth were measured using a Nikon SMZ 8 microscope set at 20×magnification; measurements were recorded to the nearest 0.01 mm. The SEM photographs of teeth were taken from uncoated specimens using a Hitachi SEM at the American Museum of Natural History.

### Nomenclatural acts

The electronic edition of this article conforms to the requirements of the amended International Code of Zoological Nomenclature, and hence the new names contained herein are available under that Code from the electronic edition of this article. This published work and the nomenclatural acts it contains have been registered in ZooBank, the online registration system for the ICZN. The ZooBank LSIDs (Life Science Identifiers) can be resolved and the associated information viewed through any standard web browser by appending the LSID to the prefix “http://zoobank.org/”. The LSID for this publication is: urn:lsid:zoobank.org:pub:7060CE3B-7EFB-4987-A8EE-50691F5D1F38. The electronic edition of this work was published in a journal with an ISSN, and has been archived and is available from the following digital repositories: PubMed Central, LOCKSS.

## Results

### Systematic paleontology

Order RODENTIA Bowdich, 1821

Family APLODONTIDAE Trouessart, 1897

Subfamily ANSOMYINAE Qiu, 1987

*PROANSOMYS*, gen. nov.

urn:lsid:zoobank.org:act:3CE4A9A6-380D-4E1E-A135-04A4BB3FF6C7

#### Type Species

**Figure 3:**
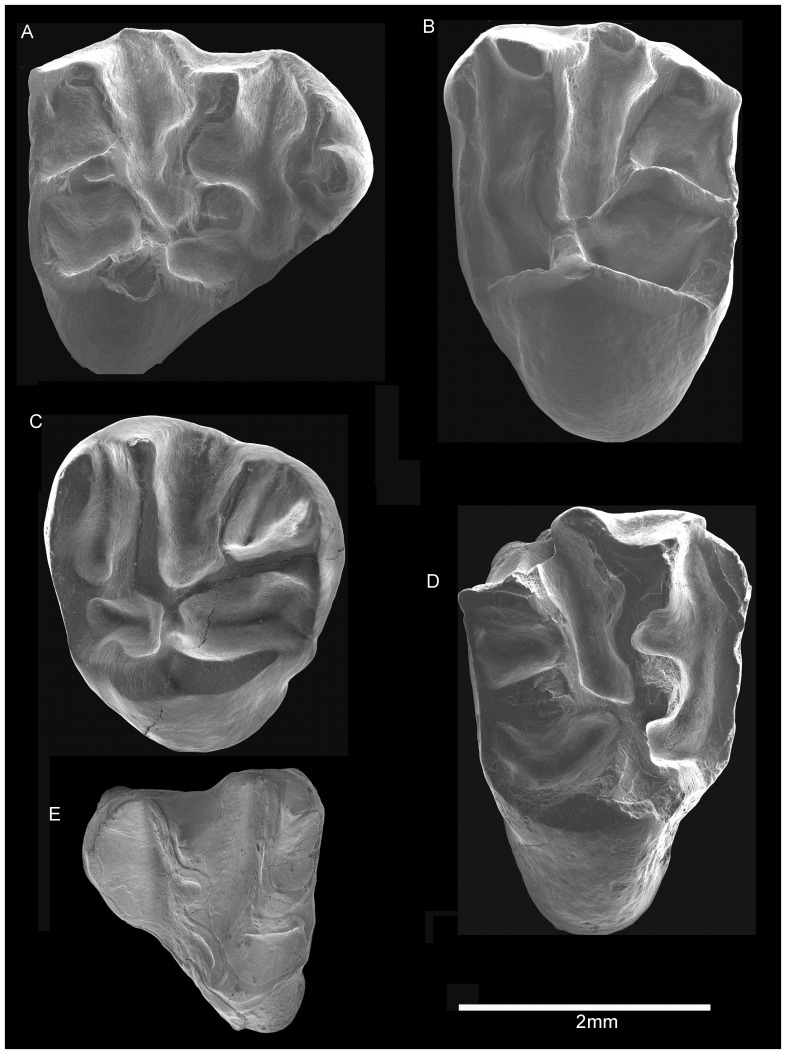
Upper dentition of *Proansomys dureensis*, sp. nov. A, IVPP V18534.3, RP4; B, V18534.5, LM1/2; C, V18533.12, LM3; D, V18537.3, RM1/2; E, V 18536.1, LDP4.

**Figure 4:**
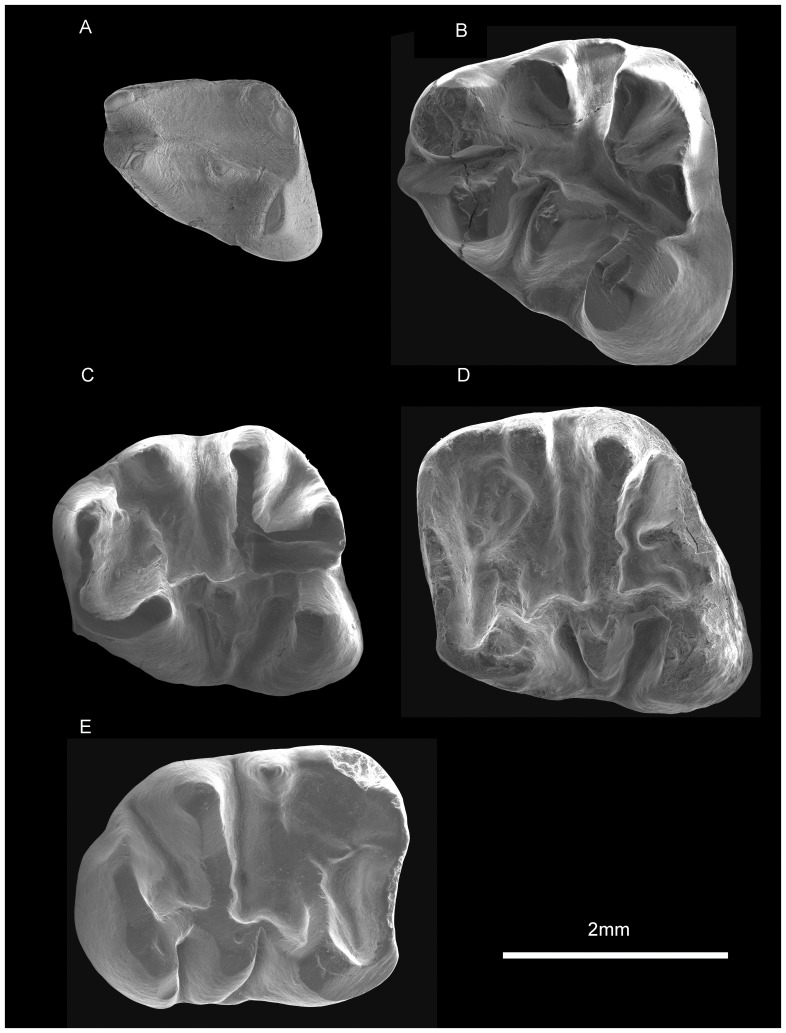
Lower dentition of *Proansomys dureensis*, sp. nov. A, IVPP V18533.19, Ldp4; B, V18534.16, Lp4; C, V18534.22, Lm1; D, V18535.1, Lm2; E, V18534.27, Rm3.

*Proansomys dureensis*, sp. nov. (Figures 3–4)

#### Diagnosis

Cheek teeth brachydont without accessory lophules; ectoloph on P4-M1/2 with a bucket-handled shape; mesostyle on P4-M1/2 single, not fully crested to close the central valley; metaconid anteroposteriorly compressed but with distinct cusp on lower molars; hypolophid attached to the hypoconulid rather than the ectolophid on p4, but complete and connected to the ectolophid on lower molars.

Differs from *Ansomys* in having a single mesostyle, a partly crested ectoloph, less developed parastyle on P4, a straight protoloph on most upper molars, weakly developed mesostylid on p4 and m1, an incomplete hypolophid on p4, a lower degree of lophodonty, and less complex tooth basins (lacking accessory lophules). Differs from *Prosciurus* in development of bucket-handle shaped ectoloph on P4 and M1/2, absence of the hypocone on upper cheek teeth, and presence of a complete hypolophid on lower molars. The cheek teeth of *Proansomys* are also generally more lophate than those of *Prosciurus*, but less lophate than those of *Ansomys*.

#### Etymology

*Pro*-, Latin “before”, implying the more primitive morphology of the new taxon in comparison with *Ansomys*.

*Proansomys dureensis* sp. nov.

urn:lsid:zoobank.org:act:631ADBC0-C9D5-4727-A0A1-FA73E71C1CE1

(Figures 3–4; Table 1)

**Table 1:** Measurements in millimeters of the teeth of *Proansomys dureensis*, sp. nov.

Tooth		Mean	Range
**dP4**	anteroposterior length	1.83	1.73–2.00
	transverse width	1.92	1.76–2.11
**P4**	anteroposterior length	2.09	1.85–2.28
	transverse width	2.19	2.17–2.62
**M1/2**	anteroposterior length	1.75	1.51–1.95
	transverse width	2.34	2.10–2.56
**M3**	anteroposterior length	1.99	1.89–2.11
	transverse width	2.15	1.99–2.41
**dp4**	anteroposterior length	1.85	1.65–2.16
	transverse width	1.54	1.41–1.66
**p4**	anteroposterior length	2.03	1.58–2.40
	transverse width	2.04	1.63–2.30
**m1**	anteroposterior length	1.95	1.85–2.11
	transverse width	1.92	1.73–2.23
**m2**	anteroposterior length	2.00	1.94–2.08
	transverse width	2.00	1.80–2.16
**m3**	anteroposterior length	2.18	1.98–2.49
	transverse width	1.78	1.65–2.03

#### Type Specimen

IVPP V18534.5, left M1 or M2 (Figure 3B).

#### Referred Specimens

Thirty-seven specimens from site XJ98023: IVPP V18533.1-3, 3 left P4; V18533.4, a right P4; V18533.5-6, 2 left M1/2; V18533.7-11, 5 right M1/2; V18533.12-16, 5 left M3; V18533.17-18, 2 right M3; V18533.19-20, 2 left dp4; V18533.21, a right dp4; V18533.22-23, 2 left p4; V18533.24-27, 4 right p4; V18533.28-29, 2 left m1; V18533.30, a left m2; V18533.31, a right m2; V18533.32-34, 3 left m3; V18533.35-37, 3 right m3. Twenty-seven specimens from site XJ98035: V18534.1; a right dP4; V18534.2; a left P4; V18534.3, a right P4; V18534.4-6, 3 left M1/2; V18534.7-9, 3 right M1/2; V18534.10-12, 3 right M3; V18534.13, a left dp4; V18534.14-15, 2 dp4; V18534.16-19, 4 left p4; V18534.20, a right fragmentary mandible with p4-m1; V18534.21, a right p4; V18534.22-23, 2 left m1; V18534.24-25, 2 left m2; V18534.26, a left m3; V18534.27, a right m3. Two specimens from site XJ20004: V18535.1, a left m2; V18535.2, a left m3. Three specimens from site XJ200208: V18536.1, a left dP4; V18536.2, a right dP4; V18536.3, a left p4. Nine specimens from site XJ200209: V18537.1, a left P4; V18537.2, a left M1/2; V18537.3-4, 2 right M1/2; V18537.5, a left M3; V18537.6, a right m1, V18537.7-8, 2 right m2; V18537.9, a right m3. One specimen from site XJ200207: V18538.1, a left fragmentary mandible with m2–m3.

#### Localities and age

XJ98023, XJ98035, XJ200208, XJ200209, and XJ20004, late Oligocene Tieersihabahe Formation; XJ200207, late Oligocene, base of the Suosuoquan Formation, northern Junggar Basin, China.

#### Etymology

*dure*, name of the town near the type locality.

#### Diagnosis

As for the genus.

### Description

All specimens are isolated brachydont teeth. At present, we prefer to interpret these specimens as from a single taxon due to small sample size, while acknowledging that some variations seen in the morphology of upper molars may indicate that more than one species is possibly present. In unworn specimens, major cusps are higher and cuspate with poorly developed lophs compared to those of *Ansomys*. The cheek teeth have smooth enamel basins, lacking accessory crests or lophules. Two small buccal roots and one strong lingual root support the upper cheek teeth. The lower molars have three roots, one under the trigonid and two under the talonid. The p4 has two roots in four specimens, and three roots in five specimens.

The P4 is triangular in outline (Figure 3A). The anterocone is large and widely separated from the protoloph by a broad valley. The parastyle and anterostyle are both present; the parastyle is much smaller than the anterocone, with the developed buccal cingulums; the anterostyle is a minute cuspule present just posterolingually at the base of the anterocone. The protocone is very prominent, with short, steep anterior and posterior arms. The conules are large relative to the buccal cusps, the protoconule being slightly larger than the metaconule. Both the protoloph and metaloph are low and zigzag shaped; the protoloph runs from the paracone to the protoconule, then bends posteriorly to converge with the metaloph buccal to the protocone. There is no hypocone. The paracone is larger than the metacone, both having a flat labial surface. The single mesostyle is connected with the paracone by a strong loph, but does not close the central valley between the metacone and the mesostyle. The ecotolph slightly bulges buccally, forming bucket-handle shaped crest.

The M1 and M2 are morphologically indistinguishable. They are sub-rectangular, much wider than long (Table 1). The anterior cingulum is strong, forming a high anterior edge of the tooth. The crescentic protocone is large with anterior and posterior crests, the crests continue with the anterior and posterior cingulums, respectively. The paracone, metacone, and metaconule are somewhat more buccolingually compressed than the counterparts of P4. The buccal surface of the paracone and metacone are flat. The protoloph is straight, running from the paracone toward the protocone, the protoconule is barely discernible and incorporated in the protoloph in eight out of thirteen specimens (Figure 3B); it is zigzagged with distinct protoconule in five out of thirteen specimens (Figure 3D). The metaloph runs from the metacone to metaconule, then bends anteriorly to converge with the protoloph just lingual to the protoconule. In ten M1/2s, the mesostyle is anteroposteriorly elongate, but not closing the central valley, whereas in three M1/2s, there is no distinct mesostyle.

The M3 is large relative to the preceding molars (Table 1). It is circular with slightly narrower posterior end (Figure 3C). Compared to M1/2, the anterior cingulum is more prominent. The posterior end of the tooth is reduced, the metacone is incorporated into the metaloph, and the metaconule is shaped as an anteroposteriorly elongate crest attached to the posterior margin. The ectoloph is not developed, and there is no mesostyle.

The DP4 is similar to P4 in morphology, but is smaller, lower-crowned, with less prominent cusps (Figure 3E). Unlike in P4, the metaloph is straight, running from the metacone toward the metaconule.

The p4 is sub-triangular in outline with tapered anterior end (Figure 4B). The protoconid and metaconid are set close together, with the metaconid distinctly higher than the protoconid. The metaconid is slightly anterior to the protoconid and is separated from the latter by a narrow notch. No anteroconid is present but there is a small cuspule at the anterior corner of the tooth on two p4s. The metastylid crest is short or absent; when present, it occurs as the posterolingual slope of the metaconid. The small mesostylid is much lower than the metaconid, with no buccal crest. The ectolophid is low, extending from the protoconid to the hypoconid, and has a large triangular mesoconid with a short buccal crest. The entoconid is located slightly anterior to the hypoconid, from which the hypolophid first extends labially, and then bends posteriorly to the hypoconulid rather than the ectolophid. The hypoconulid is distinct and separated from the hypoconid by a V-shaped posterolophid.

The m1 was preserved *in situ* in V18534.20. The posterior width of m1 is much greater than the anterior width, largely because of the posterobucally protruding hypoconid (Figure 4C). The anterior cingulum is strong, forming the anterior margin of the tooth between the protoconid and the metaconid. The metaconid, just lingual to the midline, is a distinct anteroposteriorly compressed cusp in unworn specimens, and, with wear, it is incorporated with the anterior cingulum. The metalophid crest II extends only half the distance from the protoconid to the metaconid and is absent at the metaconid side. The metastylid crest is absent. A distinct mesostylid is present posterolingual to the metaconid with a short, low crest that extends only a short distance toward the center of the tooth. The mesoconid is located at the middle of the low ectolophid and lacks a buccal mesolophid or has a very short one. The hypolophid is complete, running from the entoconid to the ectolophid posterior to the mesoconid. The hypoconulid is prominent, connected with the hypolophid by an anteriorly directed loph.

The m2 is similar to m1 but the metaconid is shifted more lingually, making it wider anteriorly than m1 (Figure 4D). The metastylid crest is more developed and joins the mesostylid along the anterior lingual border. The mesostylid is pronounced, with a longer internal crest oriented transversely that ends at about half way across the tooth. The hypoconulid is separated from the hypolophid by a narrow transversely oriented valley.

The m3 is the longest of the lower cheek teeth (Table 1). The trigonid of m3 is much like that of m2, but the posterior end is rounded and narrower (Figure 4E). The hypoconid is placed right at the posterolabial corner of the tooth, not labially protruding, unlike m1 and m2, and the hypoconulid is reduced.

The dp4 is similar to p4 in morphology, but is slender and lower-crowned, with lower cusp height (Figure 4A). The entoconid is less defined, confluent with prominent hypoconulid at the base, making the talonid basin broader.

### Phylogenetic analysis

**Figure 5:**
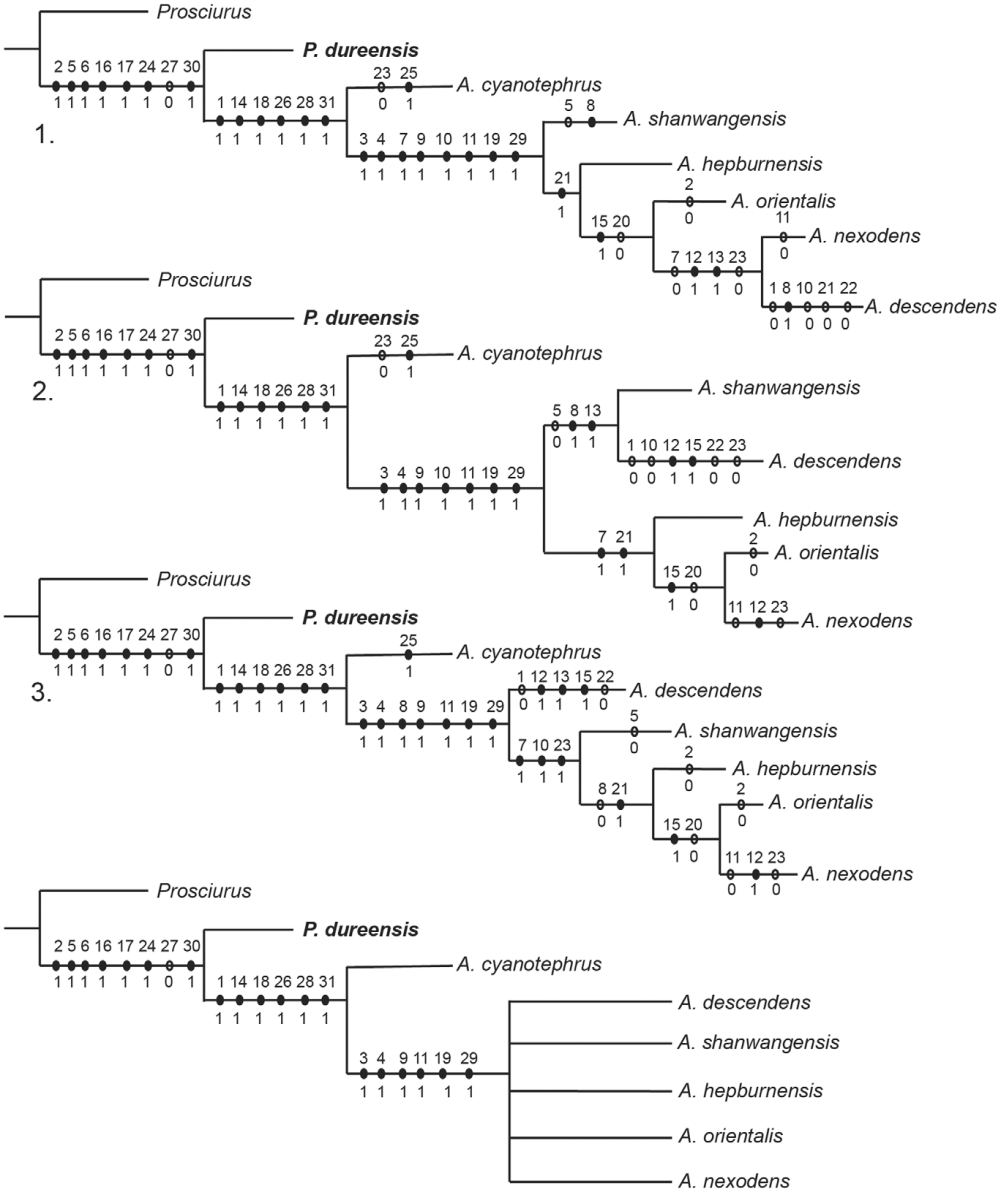
Three most parsimonious trees (MPTs 1–3) and their strict consensus tree.

To evaluate the phylogenetic position of *Proansomys dureensis*, we plot it to the character matrix of Hopkins [5] with addition of *A. cyanotephrus* [6]. *Plesispermophilus atavus* and *P. angustidens* were excluded from the analysis because they represent a different clade from the Ansomyinae (see discussion below). Additionally, *A. crucifer* and *A. shantungensis* were excluded from the analysis because they are represented by only a single tooth, respectively. *Proansomys dureensis* was coded as follows: 01111 10000 00000 11001 01110 00001 0. Heuristic searches in PAUP* version 4.0 were conducted with all characters under equal weight. 1000 replicates of random taxon addition result in three most parsimonious trees (MPTs) (Figure 5). The tree length is 42 steps, with the consistency index being 0.738 and the retention index 0.667. The strict consensus of the three most parsimonious trees is also illustrated in figure 5. *Proansomys dureensis* occurs as the basal taxon of the Ansomyinae clade in all three MPTs.

### Comparisons with species of *Ansomys*

*Ansomys shantungensis* is known only from one isolated m1 [3], so that its comparison with our samples is difficult. *Proansomys dureensis* most closely resembles *A. shantungensis* in morphology of the m1. Both taxa have an anteroposteriorly compressed metaconid, an incomplete metalophulid II, a weak mesostylid, a posterolabially extended hypoconid, and a complete hypolophid on m1. However, though *A. shantungensis* has fewer crenulations than other species of *Ansomys*, it has already begun to develop accessory lophules in the basins of the teeth. *Proansomys dureensis* has a smooth enamel basin that lacks accessory lophules.

*Proansomys dureensis* overlaps with the upper end of *A. orientalis* size range, being slightly larger [1]. They both have lophate dentition, the anterostyle is present on P4, the mesostyle is distinct, the metalophid crest II extends lingually to the center of the teeth, the metaconid is crestlike, and the hypolophid is complete on lower molars. However, the cheek teeth of *Proansomys dureensis* are more cuspate and less lophate. The occlusal pattern is very simple, lacking the complexity of accessory lophules present in *Ansomys*. The mesostyle on P4 and M1/2 is enlarged to form a bucket-handle shaped ectoloph as in *A. orientalis* but not fully crested to close the central valley. The protoloph runs straight from the paracone toward the protocone, and the protoconule is indistinguishable as part of a continuous protoloph on most upper molars. In contrast, the protoloph of upper molars is zigzag-shaped and the protoconule is distinct in *A. orientalis*. The hypolophid on dp4 and p4 is incomplete and bends posteriorly to the hypoconulid, but is complete and extends transversely to the ectolophid in *A. orientalis.* The m3 is relatively anteroposteriorly longer than that of *A. orientalis*.

*Ansomys shanwangensis* was described from the middle Miocene Shanwang Formation of Shandong Province, based on one compressed skeleton [2]. *Ansomys shanwangensis* is comparable to *Proansomys dureensis* in size, but can be easily distinguished from it. *Ansomys shanwangensis* is unique in having double protolophules, strong development of the ectoloph, developed mesoloph, complexity of accessory lophules, strong development of lophodonty, transversely elongated mesostylids on p4 and m1, and prominent entoconid on m3.

*Proansomys dureensis* is distinct from all other known species of *Ansomys* by its significantly larger size, a partly crested ectoloph, the absence of accessory lophules, lower degree of lophodonty, and the straight protoloph with crest-like protoconule on most upper molars.

## Discussion

*Proansomys dureensis* is assigned to Ansomyinae because the ectoloph of upper cheek teeth, although not fully crested, has attained the bucket-handle shaped configuration that is the most important diagnostic character of the subfamily as its name implies. Other synapomorphies uniting *Proansomys* with the Ansomyinae are P4 anterostyle doubled, labial faces of the paracone and metacone flat, lingual crest of metaconule joining protoconule, hypocone absent, m2 metaconid anteroposteriorly compressed, and basal part of hypoconid posterolabially expanded. Cladistic analysis posits *Proansomys* as the most basal ansomyine (Figure 5). It is more plesiomorphic than species of *Ansomys* in having a partly crested ectoloph on upper cheek teeth, less complex dental pattern without the accessory lophules, a lesser degree of lophodonty, metaconid on m1 lingually prominent, main cusps of lower teeth not anteroposteriorly compressed, and an incomplete hypolophid on p4. Therefore, we consider this justifies its status as a new genus.

The prosciurines have been traditionally considered a paraphyletic stem group of aplodontids that gave rise to all later aplodontids [15]. On the one hand, *Proansomys* still retains many generalized prosciurine features, such as straight protoloph with barely discernible protoconule on molars, single mesostyle, poorly developed ectoloph, the hypolophid bending posteriorly to join with posterolophid on p4, and weakly developed mesostylid on p4 and m1. On the other hand, it displays the apomorphic state of other characters (the lophodonty of the cheek teeth and development of the ectoloph on upper molars), but only to a moderate degree compared to *Ansomys* so that *Proansomys* is morphologically intermediate between *Prosciurus* and *Ansomys*. The combination of the primitive prosciurine and derived ansomyine characters in *Proansomys* suggests that the Ansomyinae derived from a prosciurine species, as did other advanced aplodontids.

*Plesispermophilus* was considered as the possible candidate for ancestral Ansomyinae [1], [5]. However, the discovery of *Proansomys* suggests that the Ansomyinae do not seem to have evolved from *Plesispermophilus* because this new species, an evident stem Ansomyinae, is more primitive than *Plesispermophilus* in having a not fully crested ectoloph and less complex tooth basin. Additionally, *Proansomys* lacks the buccal extension of mesoconid and the anterior extension of the hypoconid as in the species of *Ansomys*. In contrast, *Plesispermophilus* has a fossetid that is united with the buccal extension of mesoconid and the anterior extension of the hypoconid. This feature may also indicate that the Ansomyinae and *Plesispermophilus* represent distinct clades from each other.

*Ansomys shantungensis* was the oldest known Asian representative of the subfamily. It was recovered from a drill core in Shandong and was placed in the middle or late Oligocene on the basis of evolutionary stage as illustrated by North American and European prosciurines [3]. As mentioned above, it is most comparable to *Proansomys* in morphology of the m1, but is slightly more derived. This further indicates that *A. shantungensis* may be late Oligocene or early Miocene in age. In addition, *Proansomys* is 3,000 km northwest of *A. shantungensis*, suggesting that the subfamily was already geographically widespread during late Oligocene time.

Two alternate hypotheses have been proposed for the paleobiogeography of the *Ansomys.* Whereas Hopkins [5] proposed a European origin and a series of migrations between Europe, Asia, and North America, Korth [6] proposed an origin in North America and a single migration to Eurasia in the early Miocene. Now, recognition of the lineage (*Prosciurus*-*Proansomys*-*Ansomys*) provides new insights into the early history of the ansomyines. Although the earliest record of ansomyines was *A. cyanotephrus* from the early late Oligocene of North America [6], *Proansomys* is the most primitive member of the subfamily and appears derived from *Prosciurus*-like forms. Given that *Prosciurus* was common in the early Oligocene in Mongolia [16], the ansomyines may have originated in Asia some time prior to the late Oligocene, then dispersed throughout Eurasia and North America. The Ansomyinae may have arrived in North America prior to the early late Oligocene because *A. cyanotephrus* was known from the early late Oligocene of South Dakota. Once again, more complete fossil discoveries will be necessary to make reliable inferences about the ansomyine paleobiogeographical relations.
